# Genome-wide comparison and *in silico* analysis of splicing factor SYF2/NTC31/p29 in eukaryotes: Special focus on vertebrates

**DOI:** 10.3389/fgene.2022.873869

**Published:** 2022-09-02

**Authors:** Bao-Xing Huang, Zi-Chang Jia, Xue Yang, Chao-Lin Cheng, Xiao-Rong Liu, Jianhua Zhang, Mo-Xian Chen, Jing-Fang Yang, Yun-Sheng Chen

**Affiliations:** ^1^ Clinical Laboratory, Shenzhen Children’s Hospital, Shenzhen, China; ^2^ Co-Innovation Center for Sustainable Forestry in Southern China and Key Laboratory of National Forestry and Grassland Administration on Subtropical Forest Biodiversity Conservation, College of Biology and the Environment, Nanjing Forestry University, Nanjing, China; ^3^ State Key Laboratory of Crop Biology, College of Life Science, Shandong Agricultural University, Taian, China; ^4^ Department of Biology, Hong Kong Baptist University, State Key Laboratory of Agrobiotechnology, The Chinese University of Hong Kong, Shatin, Hong Kong SAR, China; ^5^ State Key Laboratory for Biology of Plant Diseases and Insect Pests, Institute of Plant Protection, Chinese Academy of Agricultural Sciences, Beijing, China

**Keywords:** alternative splicing, SYF2, expression profile, gene family, proteogenomics

## Abstract

The gene *SYF2*—an RNA splicing factor—can interact with Cyclin D-type binding protein 1 (GICP) in many biological processes, including splicing regulation, cell cycle regulation, and DNA damage repair. In our previous study we performed genome-wide identification and functional analysis of *SYF2* in plant species. The phylogenetic relationships and expression profiles of *SYF2* have not been systematically studied in animals, however. To this end, the gene structure, genes, and protein conserved motifs of 102 *SYF2* homologous genes from 91 different animal species were systematically analyzed, along with conserved splicing sites in 45 representative vertebrate species. A differential comparative analysis of expression patterns in humans and mice was made. Molecular bioinformatics analysis of *SYF2* showed the gene was conserved and functional in different animal species. In addition, expression pattern analysis found that *SYF2* was highly expressed in hematopoietic stem cells, T cells, and lymphoid progenitor cells; in ovary, lung, and spleen; and in other cells and organs. This suggests that changes in *SYF2* expression may be associated with disease development in these cells, tissues, or organs. In conclusion, our study analyzes the *SYF2* disease resistance genes of different animal species through bioinformatics, reveals the relationship between the *SYF2* genotype and the occurrence of certain diseases, and provides a theoretical basis for follow-up study of the relationship between the *SYF2* gene and animal diseases.

## Introduction

In eukaryotic cells, gene expression can be roughly subdivided into three steps: transcription, splicing, and translation, and is performed by RNA polymerases, spliceosomes, and ribosomes. In 1977, scientists first discovered that adenovirus mRNA and its corresponding DNA transcription template did not form a continuous hybrid double strand, but was instead an extended circular single strand DNA at different locations. This suggests that genetic information is transferred from DNA to mRNA, not only by transcription, but also by RNA splicing (Berget et al., 1977). The pre-mRNA introns and exons produced during transcription are arranged alternately. Only after intron excision and exon splicing is complete can mature mRNA be generated and enter the translation process with coherent information (Chen et al., 2012). This is known as the splicing process. RNA splicing is an important process for regulating cell differentiation, proliferation, and survival, and is equally important in gene regulation. Splicing factors participate in the splicing process of RNA precursors, and their presence causes the final protein products to show different functional and structural characteristics, thereby increasing genetic diversity. In recent years, sequencing technology and transcriptome analyses have revealed that alternative splicing is ubiquitous in various species (Chen et al., 2021; Song et al., 2021), and can lead to profound changes in gene expression patterns during development (Chen M.-X. et al., 2020). The molecular mechanism of RNA splicing consists of a two-step transesterification reaction (Toro et al., 2007; Wan et al., 2016). This deceptively simple chemical reaction is difficult to perform in cells on its own, and it requires a spliceosome to complete it. During RNA splicing, a large and highly dynamic molecular machine in the cell nucleus is pieced together from many different components. It is a ribosomal protein complex that recognizes the splicing site of the RNA precursor, and catalyzes the splicing reaction. Its size is 60 S, and it is mainly dynamically composed of a variety of non-SNRNPs, assembled small nuclear ribonucleoproteins, and RNA (Madhani & Guthrie, 1994; Will & Lührmann, 1997). It is formed at various stages of splicing with the addition of snRNA. In addition, spliceosomes are classified into major and minor spliceosomes due to differences in the proportion of intron excisions they monitor. Approximately 99.5% of intron excision reactions, which are the primary spliceosomes during splicing, are monitored. The major spliceosomes can monitor approximately 99.5% of the intron resection response, while secondary spliceosomes are less efficient in monitoring intron excision reactions, accounting for only 0.5% (Lorkovic et al., 2005; Chen & Moore, 2015). When splicing occurs, there are two steps to the assembly of the spliceosome. First, the identification of the 3′ and 5′ splice sites is completed in a base-complementary manner, and the U2 snRNP is guided to bind to the branch site to form a splice precursor. The resulting product then combines with U4, U5, and U6 snRNP trimers to form a spliceosome (Will & Luhrmann, 2011). The average human body contains approximately 100,000 spliceosomes per cell. Spliceosomes can also be classified into two types, type I and type II. The first spliceosome contains five major snRNP subcomplexes: U1, U2, and U4 to U6. Five snRNPs, U5, U11, U12, U4atac, and U6atac, constitute the type II spliceosome (Chen & Moore, 2015).

*SYF2*, also called p29, or CBPIN, or NTC31, encodes a nuclear protein. *SYF2* interacts with Cyclin D-type binding-protein 1 (GICP), is involved in cell cycle regulation and pre-mRNA splicing, and plays an important role in cancer progression.

As a cell cycle regulator, *SYF2* induces the transition of the G1-to-S phase to promote cell proliferation by interacting with cyclin-D-type binding protein 1 (Witzel et al., 2010). Cyclin D1 induces the cell cycle transition of G1-to-S phase by interacting with cyclin-dependent kinase 4/6 (CDK4/6), thereby promoting cell proliferation (Tao et al., 2020).

*SYF2* participates in the progress of diverse tumor entities, such as breast cancer (Shi et al., 2017), gastric cancer (GC) (Liu et al., 2019; Tao et al., 2020)**,** human epithelial ovarian cancer (EOC) (Yan et al., 2015), hepatocellular carcinoma (Zhang et al., 2015), esophageal squamous cell carcinoma (ESCC) (Zhu et al., 2014), and glioma (Guo et al., 2014), in a cell cycle-dependent pathway. There is a positive correlation between *SYF2* expression and proliferation of cancer, with *SYF2* a potential novel tumor marker and an oncogene. *SYF2* might potentially be the molecular target for the treatment of cancer, i.e., knocking down *SYF2* would lead to cell cycle G1/S phase arrest, and hence to inhibition of cancer cell proliferation.

*SYF2* may promote the replication checkpoint and S-phase arrest (slowdown) through both splicing-dependent and independent mechanisms. *SYF2* regulates DNA replication and cell cycle progression through AS regulation of ECT2-Ex5 (Tanaka et al., 2020). ECT2 is a protooncogene with an important role in the cytokinesis phase of the cell cycle. The ECT2-Ex5+ isoform promotes S-phase accumulation. The p29 gene is involved in intervertebral disc (IVD) degeneration (Cherif et al., 2022). *SYF2* interacts with PRP17, which is involved in the splicing and cell cycle (Ben-Yehuda et al., 2000). *SYF2* was hypomethylated in all superovulated oocytes (Huo et al., 2020).

*SYF2* is involved in many biological processes, such as splicing regulation, cell cycle regulation, and DNA damage repair. Our previous study performed genome-wide identification and functional analysis of *SYF2* in plant species (Tian et al., 2019). However, the phylogenetic relationships and expression profiles of *SYF2* in animals have not been systematically studied. In this study, we used a variety of bioinformatics methods to systematically analyze the gene structures, gene and protein motifs, and splicing conservation of the animal *SYF2* gene family. The differential expression patterns of *SYF2* in different diseases, different organs and tissues, different cells, different developmental stages, and different sampling time points in humans and mice are also discussed. This suggests that *SYF2* may be involved in the development of disease, and may be a molecular target for the treatment of cancer. This study aims to reveal the relationship between *SYF2* genotypes and biological disease processes from the perspective of bioinformatics, and to provide some basic theories for subsequent research into *SYF2* as a novel tumor marker.

## Results

### Phylogenetic tree construction of animal *SYF2* genes

**FIGURE 1:**
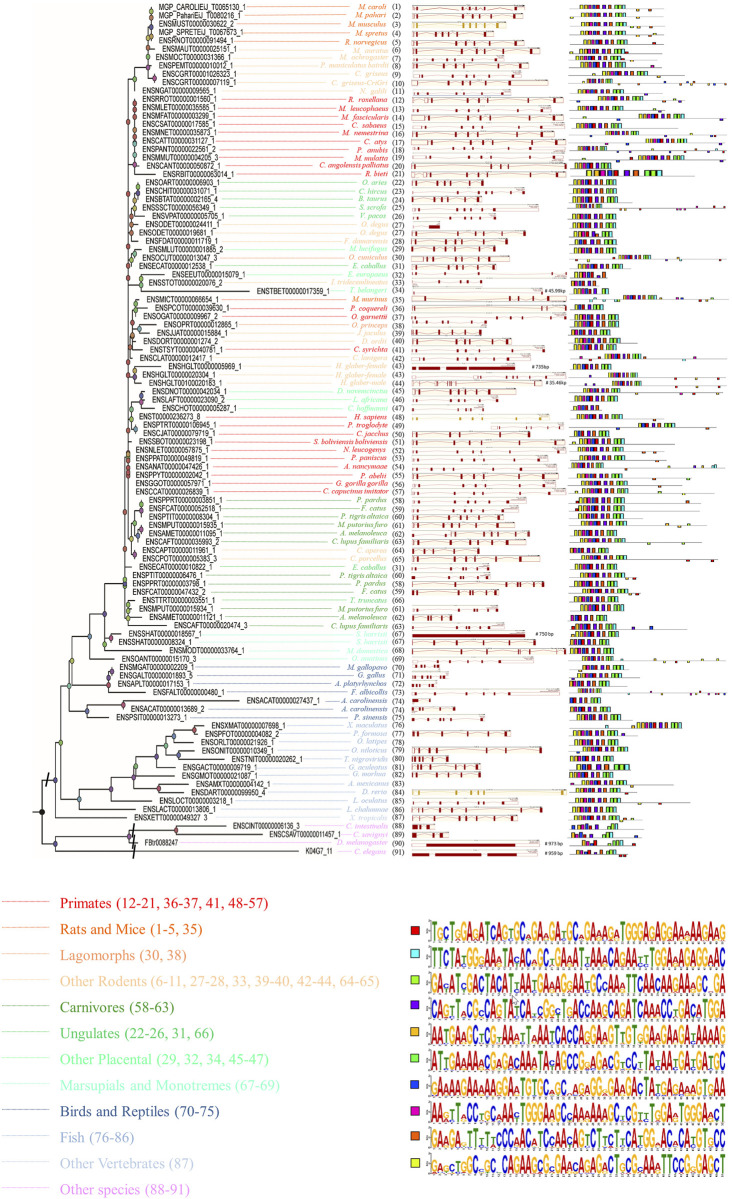
The phylogenetic relationship analysis of animal *SYF2* genes. The phylogenetic relationship is listed in the left panel. The gene structure is listed in the middle panel, and conserved motifs of cDNA sequences are listed in the right panel. The sequence of each conserved motif is listed below the phylogenetic tree.

In order to gain a deeper understanding of the function of *SYF2*, the possible *SYF2* genes in different animal species were determined according to the amino acid sequence of human *SYF2* (*Homo sapiens*, *ENST00000236273_8*). Ultimately, we found by alignment a total of 102 homologous sequences from 91 animal species, including 23 primates, 6 rats and mice, 18 other rodents, 12 carnivores, 11 fish, 8 ungulates, 7 birds and reptiles, 6 other placentals, 4 marsupials and monotremes, 2 lagomorphs, 1 other vertebrate and 4 other species (outgroup) (Supplementary Table S1). For construction of the phylogenetic tree of the *SYF2* gene, using the 102 amino acid sequence similarity of 91 animal species, see Figure 1, left. The phylogenetic tree has five main branches: primates, vertebrates, mammals, rodents and lagomorphs, and other species. These five main branches are then subdivided into twelve smaller branches. Among them, rodents and lagomorphs include rats, mice, lagomorphs, and other rodents. Other mammals include other placentals, marsupials, monotremes, carnivores, and ungulates. Other vertebrates include birds, reptiles, and fish.

### Conserved motif analysis of *SYF2*

To further study the conserved nature of *SYF2*s in animals, the gene structure, cDNA, and conserved peptide motif of *SYF2*s were analyzed. The genetic structure of *SYF2* of each animal species is shown in Figure 1 and Supplementary Table S2, which shows the number of introns and exons per sequence, and the presence of untranslated regions other than CDS. In general, the gene structure of this family is diverse, with each gene containing from 1 to 9 exons. We found different exon-intron organization in different subgroups within the same general class (Figure 1). For instance, among all *SYF2* members, there are two *degus*, whose sequences exist in a subgroup, where *ENSODET00000019681_1* has seven exons and *ENSODET00000024411_1* has only one long exon (Figure 1). Similarly, two *harrisii* exist in the same subgroup, and a similar situation exists: *ENSSHAT00000008324_1* has 6 exons, while *ENSSHAT00000018567_1* has only one long exon (Figure 1). Overall, the exon-intron distribution pattern of the *SYF2* gene varied across all animal species involved in this study and also within the same genus. It is indicated that the changes of gene structure are of great significance to the development and evolution of their gene families. Furthermore, the conserved motifs of each *SYF2* were compared and analyzed using MEME software, and it was found that 76 of the 102 sequences shared the same 10 motifs and had similar organizational structures. The remaining 26 sequences had some changes in the number or structure of conserved motifs. In addition, some sequences had less conserved motifs, thus indicating their functional diversity. The sequence of *C. angolensis palliatus* (*ENSCANT00000050872_1*), *O. degus* (*ENSODET00000019681_1*), *M. murinus* (*ENSMICT00000066654_1*), *J. jaculus* (*ENSJJAT00000015884_1*), *H. glaber-female* (*ENSHGLT00000005969_1*), *C. aperea* (*ENSCAPT00000011961_1*), *C. lupus familiaris* (*ENSCAFT00000020474_3*), *S. harrisii* (*ENSSHAT00000008324_1*), *M. gallopavo* (*ENSMGAT00000002209_1*), *A. platyrhynchos* (*ENSAPLT00000017153_1*), *A. carolinensis* (*ENSACAT00000013689_2*), *C. intestinalis* (*ENSCINT00000006136_3*), and *C. elegans* (*K04G7_11*) had nine motifs; *F. damarensis* (*ENSFDAT00000011719_1*), *T. belangeri* (*ENSTBET00000017359_1*), *P. troglodyte* (*ENSPTRT00000106945_1*), *C. jacchus* (*ENSCJAT00000079719_1*), *A. melanoleuca* (*ENSAMET00000011121_1*), *G. aculeatus* (*ENSGACT00000009719_1*), *C. savignyi* (*ENSCSAVT00000011457_1*), and *D. melanogaster* (FBtr0088247) had eight motifs; *E. europaeus* (*ENSEEUT00000015079_1*), *P. coquereli* (*ENSPCOT00000039630_1*), and *A. carolinensis* (*ENSACAT00000027437_1*) had seven motifs; *I. tridecemlineatus* (*ENSSTOT00000020076_2*) and *C. hoffmanni* (*ENSCHOT00000005287_1*) had six motifs, suggesting that the *SYF2* gene sequence is diverse in different species. Furthermore, there was no correlation between conserved motifs and gene structure after comparative analysis. For instance, *S. harrisii* (*ENSSHAT00000018567_1*) had only one long exon, but contained all 10 motifs, while *C. hoffmanni* (*ENSCHOT00000005287_1*) had six exons and five introns. These sequences had only six motifs.

**FIGURE 2:**
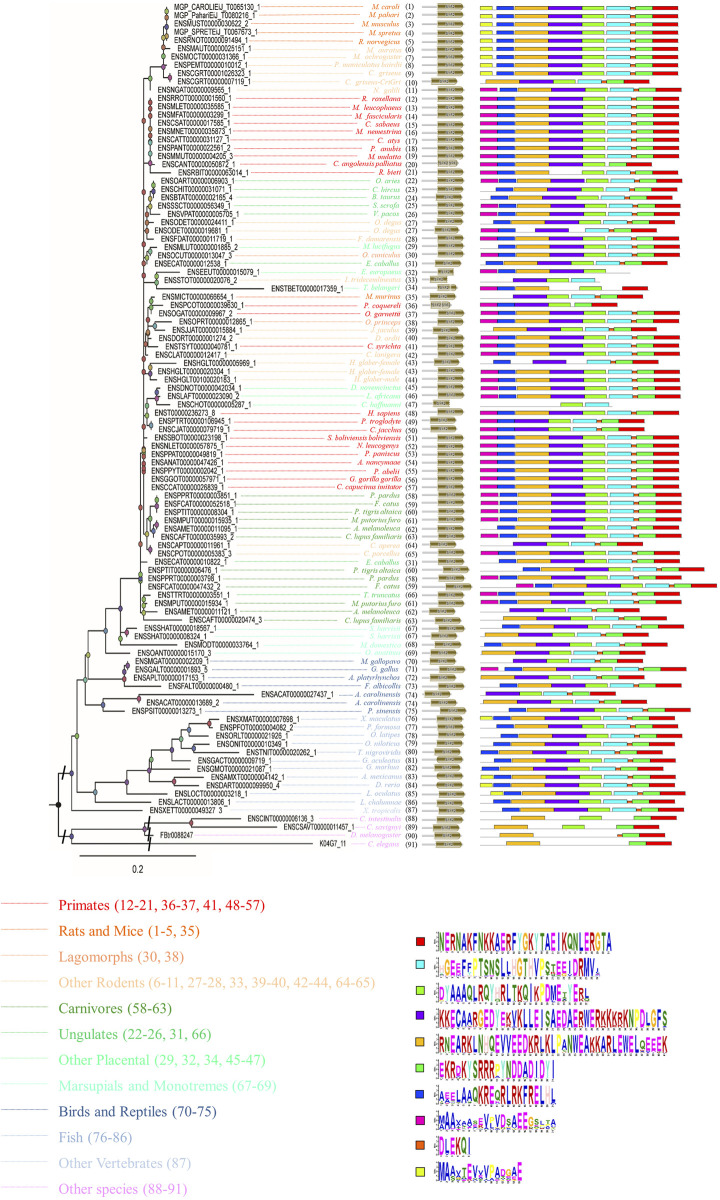
The phylogenetic relationship analysis of animal *SYF2* proteins. The phylogenetic relationship is listed in the left panel, the peptide structure is listed in the middle panel, and conserved motifs of peptide sequences are listed in the right panel. The sequence of each conserved motif is listed below the phylogenetic tree.

In addition, peptide level analysis was performed. A total of 102 sequences in different species were annotated with the *SYF2* domain (Figure 2), and MEME analysis was used to predict unknown protein motifs. Among the 102 animals belonging to different species, the animals with 8 conserved motifs accounted for the majority. Motifs shown in bright red (NERNAKFNKKAERFYGKYTAEIKQNLERGTA), pale green (DYAAAQLRQYHRLTKQIKPDMETYERL) blue (HGEEFFPTSNSLLHGTHVPSTEEIDRMV), green (EKRDKYSRRRPYNDDADIDYI), purple (KKECAARGEDYEKVKLLEISAEDAERWERKKKRKNPDLGFS), ginger (RNEARKLNHQEVVEEDKRLKLPANWEAKKARLEWELQEEEK), and dark blue (AEELAAQKREQRLRKFRELHL) occur in nearly every one of them, accounting for 70% of all motifs analyzed in this paper. Furthermore, the rose red motifs (MAAXAASEVPVDSAEEGSLTA) were concentrated in primate sequences, and only sparsely distributed in sequences of other animal species. The yellow motif (MAAXTEVVVPADGAE) only existed in rat, mouse, fish, and other rodent sequences, but was not detected in other animal sequences. In summary, through the analysis of conserved motifs at the RNA/cDNA and protein levels, it can be found that there is little difference in codon usage among *SYF2* orthologs, and the number and position of motifs are obviously similar. This indicates that *SYF2* is highly conserved among different proteins and cDNAs in different animal species.

### Construction of the *SYF2* protein interaction network

**FIGURE 3:**
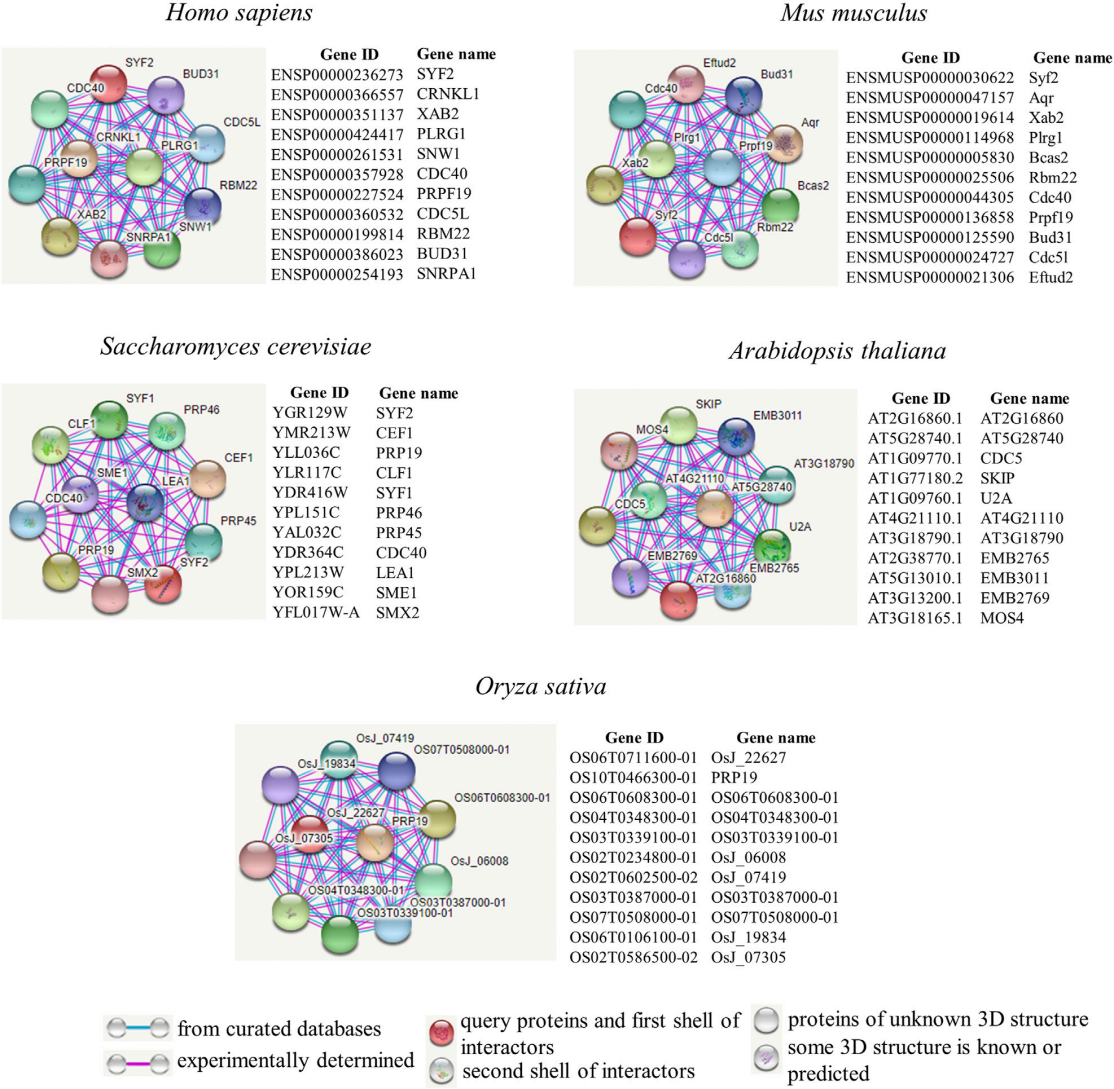
Protein interaction network diagrams of representative animal and plant species. Known interactions, either determined by experiments (pink line), or from curated databases (blue line), are presented in protein–protein interaction networks. *SYF2s* of *Homo sapiens*, *Mus musculus*, *Saccharomyces cerevisiae*, *Arabidopsis thaliana,* and *Oryza sativa* were used as query proteins for analysis by the STRING database. Highly scored interactors are presented in the form of a network diagram. Empty notes are proteins with unknown 3D structures, while filled notes are proteins with known or predicted 3D structures in the current database.

We further explored how *SYF2* plays a role in biological regulatory processes. Next, we employed the tool STRING to construct a protein interaction network of *SYF2* in different species (Figure 3). We used protein intercrops to interact with *SYF2* in organisms, including two animal species (*Homo sapiens*, *Mus musculus*), yeast, and two plant species (*Arabidopsis* and *Oryza sativa*).

Human XAB2 protein is a protein interactor of *SYF2*. It consists of 15 repeated tetrapeptides, and was identified by interaction with xeroderma pigmentosa histone A (XPA) (Nakatsu et al., 2000; Kuraoka et al., 2008). It is a novel component involved in transcriptional coupling repair and transcription, and plays a role in mitotic cell cycle regulation (Hou et al., 2016). Furthermore, there is also a clear interaction between CDC40 and *SYF2*. The CDC40 (PRP17) gene in *S. cerevisiae*, whose mutation results in sensitivity to temperature changes, was originally identified in CDC40-1 (Orna & Martin, 2002). It plays a variety of roles in cell cycle progression. Its mutation causes the cell cycle to stall (Kaplan & Kupiec, 2007). In addition, Cdc40p, Slu7p, Prp22p, Prp18p, Prp16p, and Prp8p acted as pre-mRNA splicing factors during the second splicing reaction stage (Vijayraghavan et al., 1989; Schwer & Gross, 1998).

In the mouse protein interaction map, we also found proteins with high interaction with *SYF2*. Prpf19 is a functionally diverse protein (Yin et al., 2012), is highly conserved, and participates in splicing as a splicing factor (He et al., 2021).

### Analysis of transcript isoforms and conserved splice sites

**FIGURE 4:**
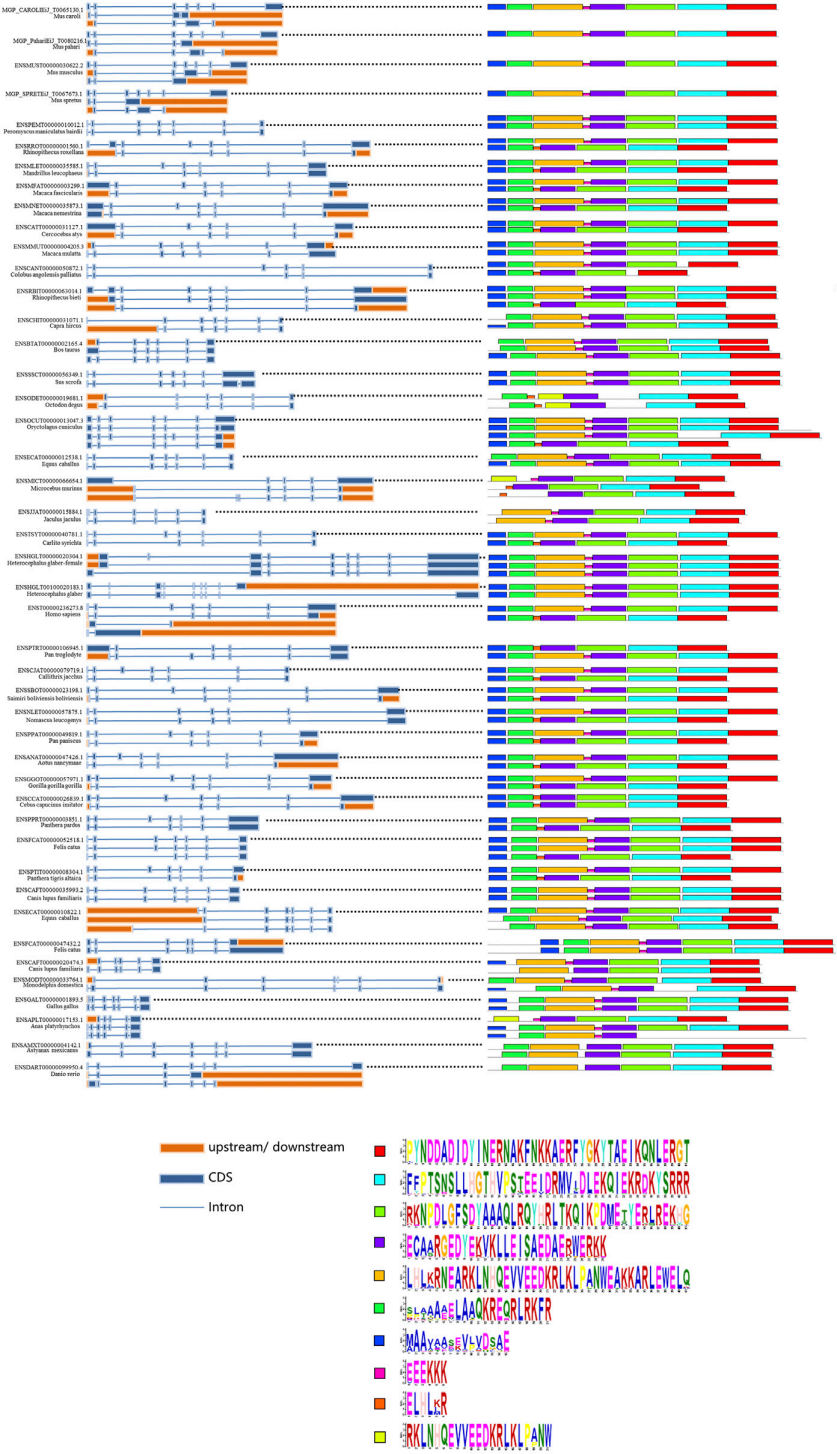
Summary of splice isoforms of the animal *SYF2* gene. Transcript isoforms from 45 animal SYF2 genes are summarized (left and middle panel). Conserved protein motifs and sequences of potential protein products from splicing isoforms are illustrated (right panel and bottom of the figure, respectively), with additional annotation to define exon–exon boundaries (blue lines between boxes).

To further understand the splicing patterns and conserved splicing sites of the *SYF2* gene, we extracted some available animal *SYF2* gene transcription subtypes from the Ensembl database and then selected 45 representative animals for alternative splicing analysis (Figure 4). A total of 106 splice isomers were obtained from 45 *SYF2* genes, with an average of 2–3 transcripts per gene. Among them, the human and *Oryctolagus cuniculus SYF2* genes annotated 4 subtypes, the largest number of annotated subtypes in these animals. When comparing the conserved motifs of the transcription subtype with the genome structure (Figure 4, right), it was found that the original transcripts of most genes contained the most motifs, while the remaining replacement transcripts usually contained fewer motifs. Tthe variable splicing event was then analyzed. First, in *Mandrillus leucophaeus*, *Macaca fascicularis*, *Macaca nemestrina*, *Cercocebus atys*, *Colobus angolensis palliatus*, *Carlito syrichta*, *Callithrix jacchus*, *Saimiri boliviensis boliviensis*, *Pan paniscus*, *Aotus nancymaae*, *Gorilla gorilla*, *Cebus capucinus imitator*, *Panthera pardus*, *Felis catus*, *Panthera Tigris altaica*, etc., there was a large amount of exon skipping. Second, an in-depth study found that the number of alternative splicing events of the first exon and the last exon (AFE and ALE), accounted for the bulk of the total alternative splicing events in *SYF2*, which indirectly led to the generation of many truncated transcript subtypes, such as in *Mus caroli*, *Mus pahari*, *Rhinopithecus roxellana*, *Macaca fascicularis*, *Cercocebus atys*, *Rhinopithecus bieti*, *Microcebus murinus*, *Gorilla gorilla gorilla*, *Oryctolagus cuniculus,* and so on. Moreover, alternative transcription initiation and alternative polyadenylation of several transcripts have been found, such as in *Capra hircus*, *Microcebus Murinus*, and *Danio rerio*.

### Analysis of the *SYF2* expression profile in animals

**FIGURE 5:**
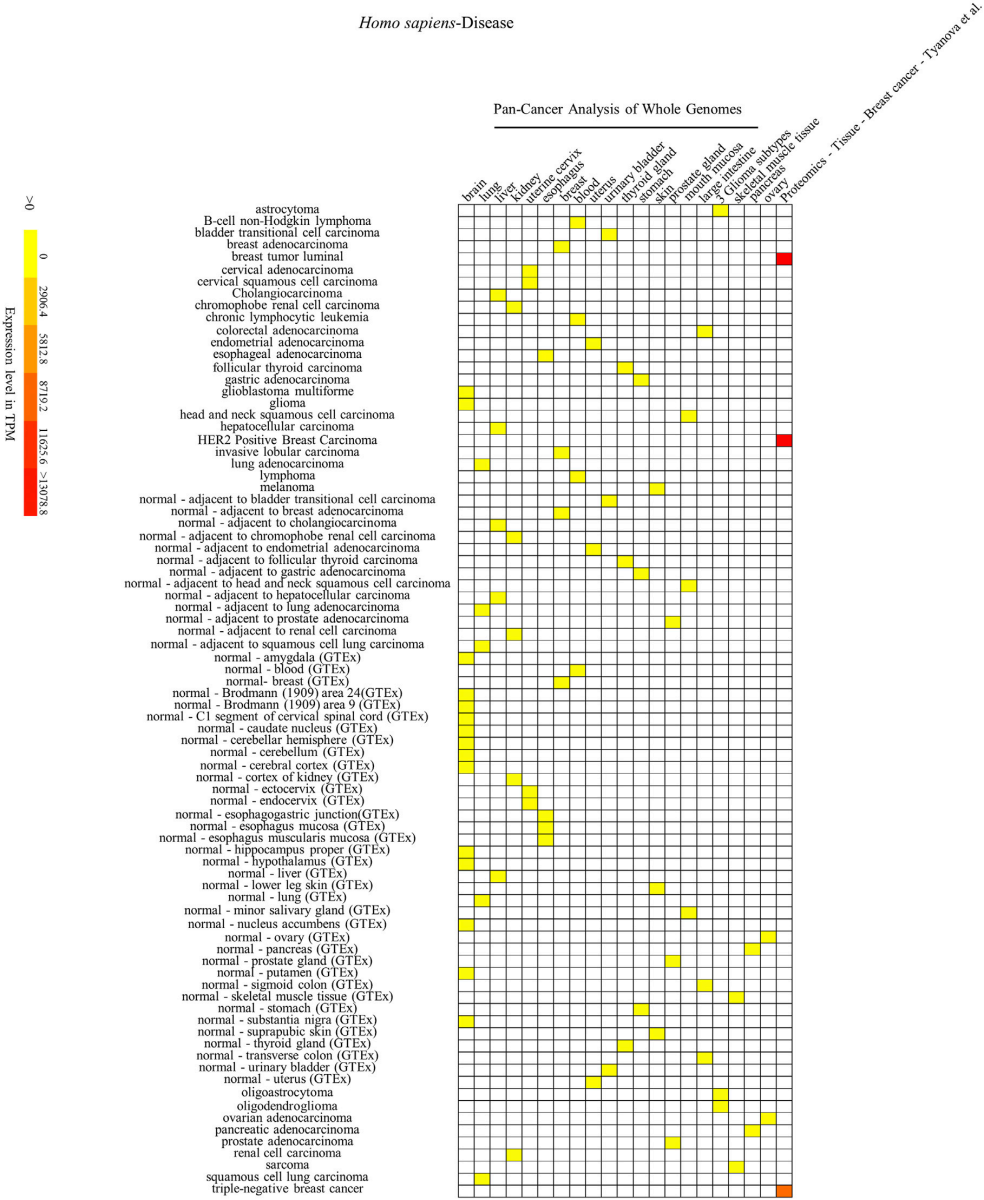
Heatmap of *Homo sapiens*’ SYF2 gene expression in different diseases. Disease database is from the International Cancer Genome project: Pan-Cancer Analysis of Whole Genomes (PCAWG). GTEx means Genotype-Tissue Expression (GTEx) Project. No. 1–21 represent 21 data source projects, which are Pan-Cancer Analysis of Whole Genomes – (brain, lung, liver, kidney, uterine cervix, esophagus, breast, blood, uterus, urinary bladder, thyroid gland, stomach, skin, prostate gland, mouth mucosa, large intestine) (1–16), 3 Glioma subtypes (17), Pan-Cancer Analysis of Whole Genomes – (skeletal muscle tissue, pancreas, ovary) (18–20), Proteomics – Tissue – Breast Cancer – Tyanova (21), respectively. Baseline expression levels are in TPM (transcripts per million). The raw data were reorganized and presented as heatmaps using online BAR HeatMapper Plus software (http://bar.utoronto.ca/ntools/cgi-bin/ntools_heatmapper_plus.cgi).

**FIGURE 6:**
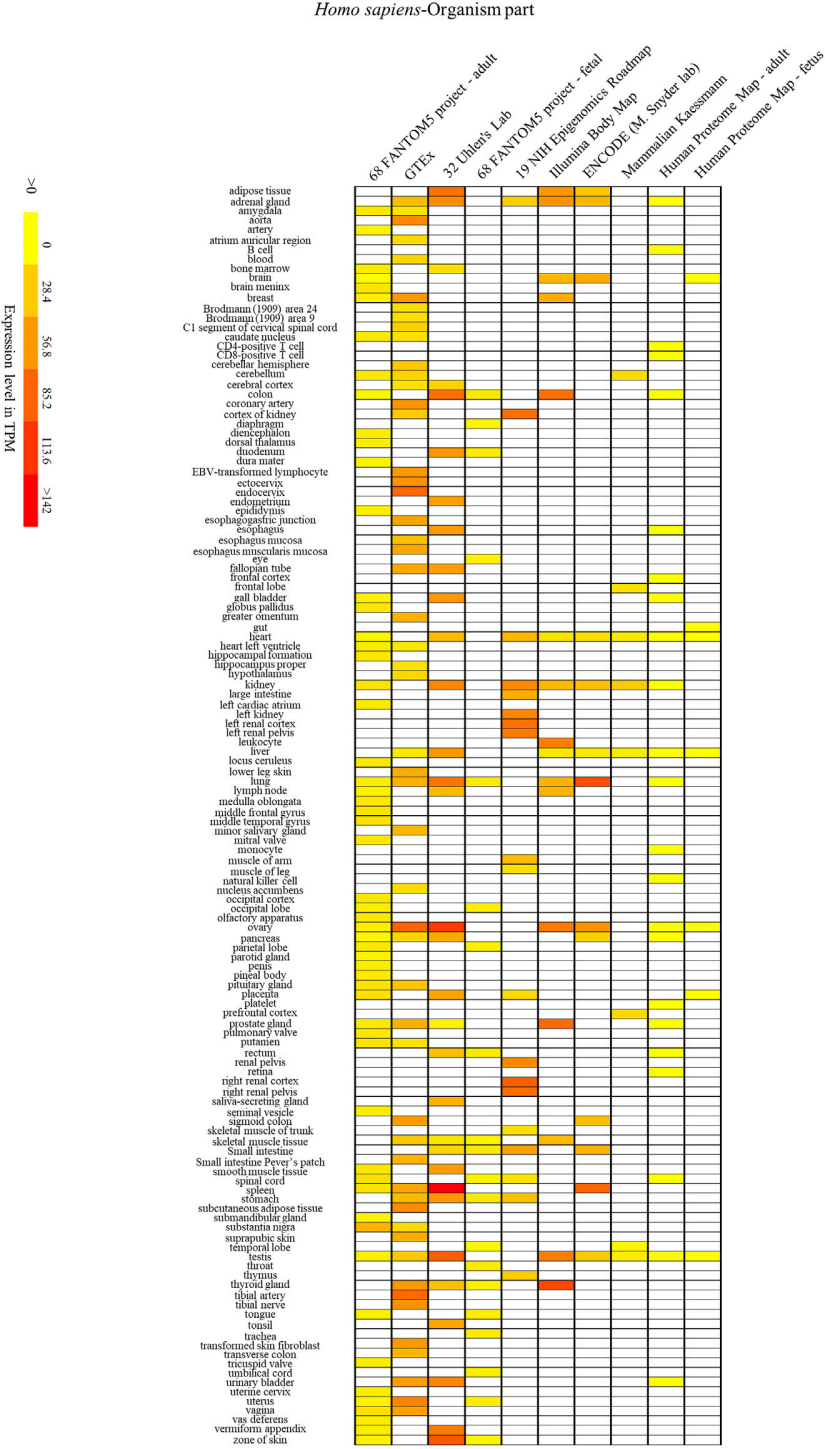
The human SYF2 gene is specifically expressed in different organs. Nos. 1–10 represent 10 data source projects, which are 68 FANTOM5 project – adult (1), GTEx (2), 32 Uhlen’s Lab (3), 68 FANTOM5 project – fetal (4), 19 NIH Epigenomics Roadmap (5), Illumina Body Map (6), ENCODE (M. Snyder lab) (7), Mammalian Kaessmann (8), Human Proteome Map – adult (9), Human Proteome Map – fetus (10), respectively. Baseline expression levels are in TPM (transcripts per million). The raw data were reorganized and presented as heatmaps using online BAR HeatMapper Plus software (http://bar.utoronto.ca/ntools/cgi-bin/ntools_heatmapper_plus.cgi).

**FIGURE 7:**
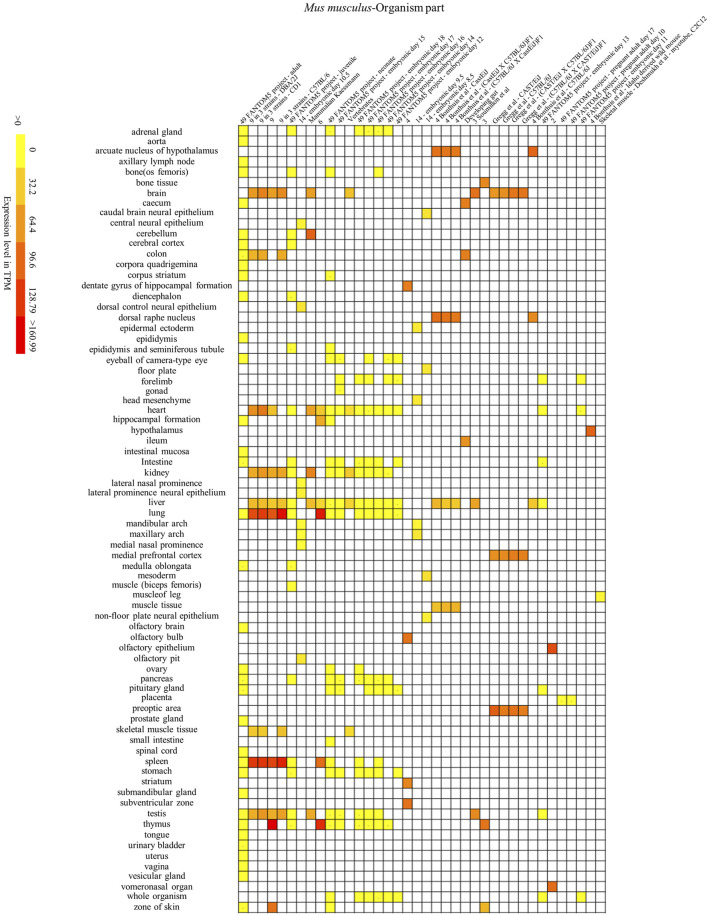
Mouse *SYF2* is specifically expressed in different organs. Nos. 1–38 represent 38 data source projects, which are 49 FANTOM5 project – adult (1), 9 in 3 strains – (DBA/2J, CD1) (2–3), 9 (4), 9 in 3 strains - C57BL/6 (5), 49 FANTOM5 project – juvenile (6), 14 – embryonic day 10.5 (7), Mammalian Kaessmann (8), 6 (9), 49 FANTOM5 project – (neonate, embryonic day 15) (10–11), Vertebrates (12), 49 FANTOM5 project – (embryonic day 18, 17, 16, 14, 12) (13–17), 4 (18), 14 – (embryonic day 9.5, 8.5) (19–20), 4 Bonthuis et al. – (CastEiJ, (CastEiJ X C57BL/6J) F1), (C57BL/6J X CastEiJ) F1) (21–23), Developing gut (24), 3 Soumillon et al. (25), 3 (26), Gregg et al. – (CAST/EiJ, C57BL/6J, (CAST/EiJ X C57BL/6J) F1, (C57BL/6J X CAST/EiJ)F1) (27–30), 4 Bonthuis et al. – C57BL/6J (31), 49 FANTOM5 project – embryonic day 13 (32), 2 (33), 49 FANTOM5 project – (pregnant adult day 17, 10, embryonic day 11) (34–36), 4 Bonthuis et al. – Idaho derived wild mouse (37), Skeletal muscle – Deshmukh et al. – myotube, C2C12 (38), respectively. Baseline expression levels are in TPM (transcripts per million). The raw data were reorganized and presented as heatmaps using online BAR HeatMapper Plus software (http://bar.utoronto.ca/ntools/cgi-bin/ntools_heatmapper_plus.cgi).

To further investigate the association between the animal *SYF2* gene and certain cell, tissue, and organ diseases, we analyzed the expression patterns of the *SYF2* gene in different animal species. Mice have genome sequences that are highly similar to humans, with gene homology as high as 78.5%. In addition, mice are close to humans in terms of biological evolution, their tissue and organ structure and cell functions are similar to those of humans, while their placenta formation and early embryonic development are also similar to humans. Therefore, we performed a comparative analysis of *SYF2* gene expression patterns in humans and mice. Through the BAR HeatMapper Plus tool, we reconstructed the expression profile to include three aspects: human disease (Figure 5), human and mouse tissues and organs (Figures 6 and 7), and human and mouse cell types and development stages (Supplementary Figures S1–S3).

First, accumulation of *SYF2* was found in breast tumor lumen, triple-negative breast cancer, and HER2-positive breast cancer (Figure 5). Second, based on multiple datasets, *SYF2*s were highly expressed in spleen and lung tissue in both humans and mice (Figures 6 and 7). However, there are also differences in the locations and levels of *SYF2* expression between humans and mice. Human *SYF2* accumulates specifically in the reproductive organs, bladder, thyroid, and colon (Figure 6), while mouse *SYF2* is abundant in tissues such as the cerebellum and thymus (Figure 7). Third, analysis of cell-type expression profiles showed that in humans, *SYF2* is high in common lymphoid progenitors and hematopoietic stem cells. By contrast, mouse *SYF2* accumulates in both native thymus-derived CD4-positive αβ T cells and induced T regulatory cells (Supplementary Figures S1 and S3). Moreover, *SYF2* was expressed at high levels in mice of different strains, and at different sampling times, and different developmental and somite stages (Supplementary Figure S2). The developmental map showed a high abundance of human *SYF2* in both the fetal and juvenile stages (Supplementary Figure S1). Across the developmental stages in mice, the accumulation of *SYF2* was higher in two stages—the embryonic stage and a few days after parturition (Supplementary Figure S2). In summary, the expression patterns of *SYF2* in humans and mice are not consistent, indicating that different species have different expression patterns due to the existence of different transcription and translation patterns. However, the study of *SYF2* in different species will help to reveal more possible regulatory roles of *SYF2*, which is conducive to the further analysis of *SYF2* function. Comparative analysis of expression patterns in humans and mice can help provide a theoretical basis for research into, and the treatment of some diseases. The comparison of human and mouse *SYF2* gene expression patterns across tissues, cell types, and developmental stages is summarized in Table 1.

**TABLE 1:** Comparison of human and mouse *SYF2* gene expression patterns in tissues, cell types and developmental stages.

	Human	Mouse
	Reproductive organs	The cerebellum
Tissues	Bladder	Thymus
	Thyroid and colon	
Cell-type expression profiles	Lymphoid progenitors	Native thymus-derived CD4-positive αβ T cells
	Hematopoietic stem cells	Induced T regulatory cells
Developmental stages	Fetal stages	The embryonic stage
	Juvenile stages	A few days after parturition

## Discussion

### Phylogenetic and splicing pattern analysis indicates *SYF2* conserved among animals

Numerous existing reports suggest that 15–35% of human disease is caused by mis-splicing or mis-assembly of spliceosome complex proteins (Shi, 2017). However, underlying evidence for how mis-splicing causes disease is lacking. Therefore, understanding the underlying mechanisms of splicing regulation will not only contribute to the decoding of the eukaryotic splicing machinery, but may also provide new targets for clinical drug discovery. We performed phylogenetic and splicing pattern analyses of *SYF2* in this work to reveal its structural conservation and potential regulatory mechanisms across different animal species.

Phylogenetic topology shows that *SYF2* proteins can be divided into 12 groups: primates, rats and mice, lagomorphs, other rodents, carnivores, ungulates, other placentas, marsupials and monotremes, birds and reptiles, fish, other vertebrates, and other species. In this, all vertebrate species are aggregated into one large group, showing distant relationships with other species, such as *Ciona intestinalis*, *Ciona savignyi*, *Caenorhabditis elegans*, and *Drosophila melanogaster* (Figure 1). Furthermore, animal *SYF2* were subjected to conserved splicing pattern analysis (Figure 4). Similar to *SYF2* previously reported in plants (Tian et al., 2019), truncated transcripts exist for the animal *SYF2* gene, resulting in the creation of a conserved protein form with N-terminal truncation (Figure 4). Splice site analysis revealed that AFE and ALE were the most prominent AS events in the numerous animal species involved. In terms of the number of transcript isoforms, the *SYF2* gene in different animal species generally has more than one transcript isoform, but it is worth noting that each transcript isoform has a similar structure, suggesting that they have similar functions in the regulation of gene expression. Moreover, most protein isoforms corresponding to each transcriptional isoform were considered functional. Therefore, the relevant biological functions of *SYF2* protein isoforms in animal species need further study.

### Differential expression patterns of animal *SYF2*s reveal functional diversity

*SYF2* induces a transition from the G1 to S phase to promote cell proliferation, and does so by interacting with the cyclin-D-type binding protein 1 (Witzel et al., 2010). Embryonic and juvenile stages are important periods of cell growth and development in the developmental cycle of animals. Human and mouse expression profiling data indicate significant enrichment of *SYF2* both in human fetal and juvenile stages, and in mouse embryonic and postpartum periods. Previous studies have also shown that disruption of *SYF2* in mice leads to embryonic lethality (Chen et al., 2012). In *Arabidopsis*, high enrichment of *SYF2* was clearly detected in the shoot apex during flowering transformation, but decreased enrichment and repressed expression of *SYF2* were found in more mature pollen (Tian et al., 2019). This suggests that *SYF2* can participate in embryonic developmental regulation by mediating cell cycle regulation.

The regulation of the cell cycle by *SYF2* is also associated with the occurrence of many cancers. In the detection of disease expression profiles, *SYF2* was significantly expressed in breast tumor lumen, triple-negative breast cancer, and HER2-positive breast cancer (Figure 5). In addition, in the organ-tissue-related expression profiles, *SYF2* was enriched in the human ovary, testis, spleen, lung, bladder, thyroid, and colon (Figure 6), as well as in the mouse cerebellum, thymus, spleen and lung (Figure 7). Among these, many organ diseases caused by abnormal expression of *SYF2* have been confirmed. For example, overexpression of *SYF2* affects the cell cycle or cell proliferation leading to the occurrence and progress of breast cancer (Shi et al., 2017), non-small cell lung cancer (Liu et al., 2015; Chen et al., 2019), and ovarian cancer (Yan et al., 2015).

Interestingly, the comparative analysis of human and mouse expression data revealed that *SYF2* expression patterns were different in different cells, tissues, organs, and developmental stages of humans and mice. These results indicate that the regulatory patterns of transcription and translation vary by species, although this is not absolute. Some similarities have been detected in expression in certain organs and tissues. For instance, high expression of *SYF2* was detected in organs such as the lung and spleen of both humans and mice (Figures 6 and 7). These results provide new entry points for the treatment of certain organ diseases.

In this article, we compared and analyzed the expression patterns of humans and mice, and summarized the experimental data in each project by means of bioinformatics. We offered preliminarily speculation on the possible function of *SYF2*, which is expected to provide a direction and a theoretical basis for research into clinically relevant diseases. Analysis of results may be affected by different experimental and sampling conditions between projects. However, modern SWATH-MS proteomics technology (Chen M. X. et al., 2020; Shen et al., 2021) could be used to study the potential function of *SYF2* further, and to verify the existing analysis results. It would be helpful to explore expression of the potential function of *SYF2*, and in so doing create more possibilities for the treatment of diseases caused by its abnormal expression.

### Comparison of *SYF2* in animals, yeast, and plants

The *SYF2* in animals, yeast, and plants (*Arabidopsis*, *Oryza sativa*) was compared. First, by analyzing the interaction network, it was found that there were only 1–2 common interacting proteins among the three species, which indicates that *SYF2* has specific regulatory networks in animals, plants, and yeast (Figure 3). Second, transcriptional isoforms of *SYF2* averaged 2–3 in animal species (Figure 4), with only one copy in most yeast and plants (Tian et al., 2019). This suggests that *SYF2* may play a more important role in animal species. The functional roles of *SYF2* in these three species are conserved to some extent.

## Conclusion

Throughout the study, we screened 102 *SYF2* genes in 91 animal species and analyzed their phylogeny, gene structure, gene and protein motifs, conservation of splicing patterns, and expression patterns. Analysis of related structures, motifs, and splicing patterns showed that *SYF2* is highly conserved in many animal species. In addition, the analysis of expression patterns showed that *SYF2* is associated with the occurrence of cancer in breast, lung, spleen, and reproductive organs, as well as other diseases. These results are intended to help reveal the possible relationship between the *SYF2* genotype and the occurrence of certain diseases, which can provide information about subsequent *SYF2* expression in studies where animals provide the basis.

## Materials and methods

### Identification and screening of *SYF2* protein sequences in animals

In the Ensembl database (http://asia.ensembl.org/), protein BLAST was performed based on the *Homo sapiens SYF2* protein sequence (ENST00000236273_8) as a template. All available gene sequences were found in animal genomes, and further screening was performed through HMMER 3.2.1 (Johnson et al., 2010).

### Construction of the *SYF2* gene phylogenetic tree in animals

A phylogenetic tree was constructed using the protein sequences of 102 *SYF2* genes obtained from the Ensembl database. Where a gene had more than one transcript, the longest coding sequence was selected. Selected sequences were subjected to comparative analysis by Muscle V3.8 (Edgar, 2004), after which a root phylogenetic tree was constructed using maximum likelihood implemented in PhyML V3.03730 (Gascuel, 2010). Finally, FigTree V1.4.3.3831 was used to edit and present the phylogenetic tree (Morariu et al., 2008). The reliability of the phylogenetic tree was tested by bootstrapping repeated sampling. Nucleotide sites were randomly selected from the original sequence to form a new set of gene sequences, and the same method was used to construct another phylogenetic tree. The topology of this phylogenetic tree was repeatedly compared with the structure of the original tree. Internal branches of the original phylogenetic tree with the same sequence separation as the bootstrap value were assigned a value of 1, while other internal branches were assigned a value of 0. We calculated the percentage of eigenvalue 1 obtained for each internal branch of the original phylogenetic tree to verify the reliability of the phylogenetic tree (Katsura et al., 2017; Fan et al., 2021).

### Analysis of gene structures, protein domains and MEME motifs

All necessary *SYF2*-related gene and protein sequence information, as well as intron and exon structure information, was downloaded from Ensembl. Subsequently, the Gene Structure Display Server 2.0 (http://gsds.gao-lab.org/) was used to reconstruct gene structure (Hu et al., 2014). The HMMER website (https://www.ebi.ac.uk/Tools/hmmer/) was used to predict the protein structure domain (Potter et al., 2018). The cDNA and amino acid sequences of all the screened genes were entered into the MEME (https://meme-suite.org/meme/tools/meme), and the 10 most conserved motifs corresponding to the sequences were systematically predicted and analyzed.

### Construction of protein interaction networks

The interacting proteins of *Homo sapiens* (ENST00000236273_8), *Mus musculus* (ENSMUST00000030622_2) and *Saccharomyces cerevisiae* (YGR129W) were analyzed through the STRING online database (https://string-db.org/), and the proteins with high interaction rankings were presented through the protein–protein interaction network. Finally, predicted functional partners (confidence cutoff of 0.900) of *SYF2* proteins were presented in the form of an interaction network drawn by Cytoscape 3.8 software.

### Analysis and identification of conserved splicing profiles and splice sites

Useful splice isomer sequences for the *SYF2* gene were collected from Ensembl. The cDNA sequence information corresponding to the gene was entered into the MEME to obtain the corresponding motif information for each transcript.

### Analysis of *SYF2* expression by online microarray datasets

The required *SYF2* expression data were downloaded through the Expression Atlas (https://www.ebi.ac.uk/gxa/home). The online BAR HeatMapper Plus software (http://bar.utoronto.ca/ntools/cgi-bin/ntools_heatmapper_plus.cgi) (Chen M. X. et al., 2020) was then used to rearrange the obtained original data as required, before finally presenting it in the form of a heatmap.

## Data Availability

The datasets presented in this study can be found in online repositories. The names of the repository/repositories and accession number(s) can be found in the article/Supplementary Material.
