# Supplementary material for: Genome-wide comparison and in silico analysis of splicing factor SYF2/NTC31/p29 in eukaryotes: Special focus on vertebrates
Source: Front Genet. 2022 Sep 2;13:873869. doi: 10.3389/fgene.2022.873869 (PMC9479762; doi:10.3389/fgene.2022.873869)
Supplement: Supplementary file 3 [file DataSheet1.docx]

**
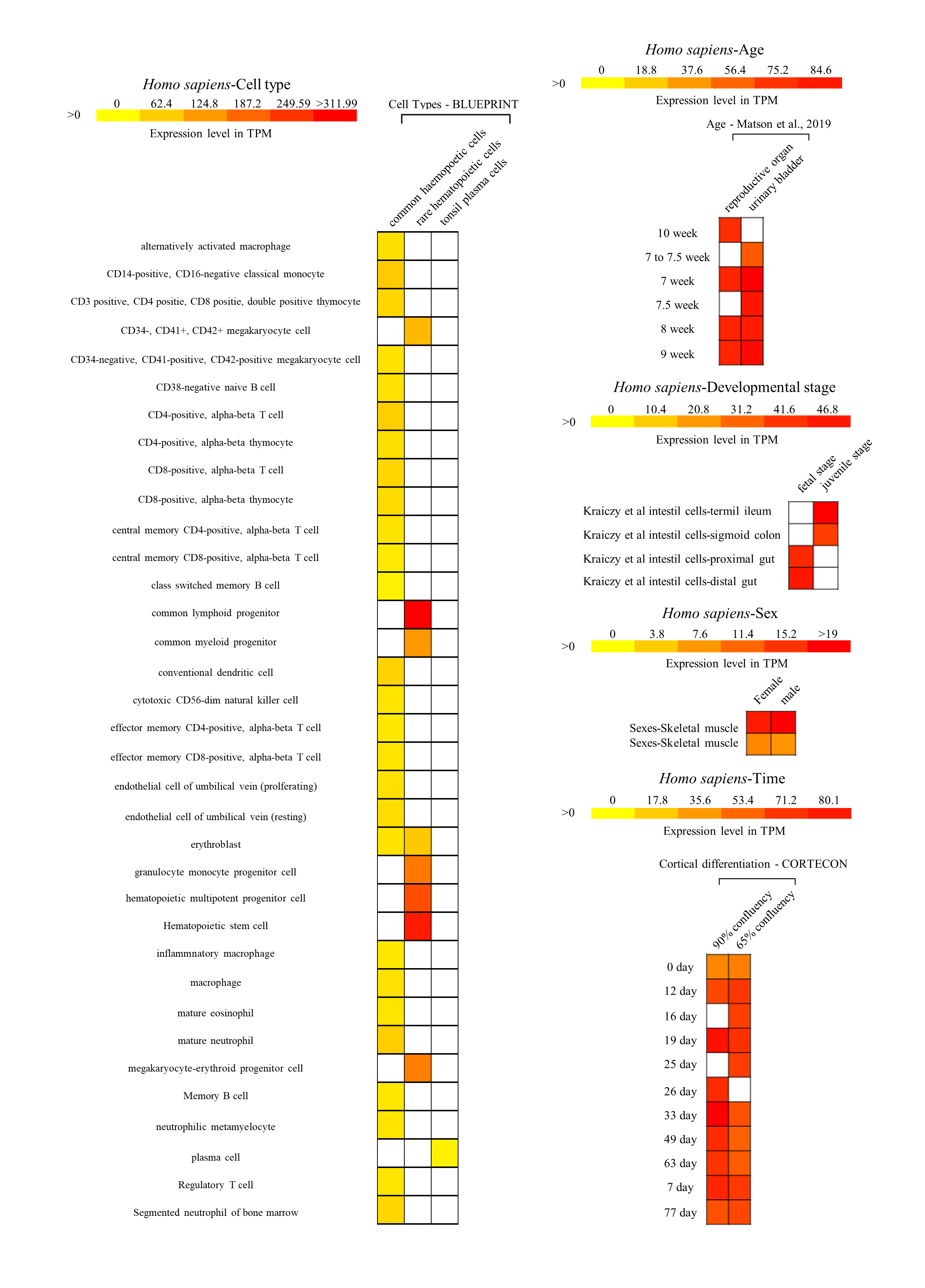
**

**Fig. S1.** **Expression of human SYF2 in various cell types, ages, developmental stages, sexes and times.** Homo sapiens-cell type Nos. 1-3 represent 3 data source projects, which are cell types - (BLUEPRINT common haematopoietic cells, BLUEPRINT rare haematopoietic cells, BLUEPRINT tonsil plasma cells) (1-3). Homo sapiens-age No. 1 and 2 represent 2 data source projects, which are Age - Matson et al., 2019 – (reproductive organ, urinary bladder) (1-2). Homo sapiens developmental stage No. 1-2 represent 2 data source projects, which are the foetal stage (1) and juvenile stage (2). Homo sex Nos. 1 and 2 represent 2 data source projects, which are female (1) and male (2). Homo sapiens-time No. 1-2 represent 2 data source projects, which are cortical differentiation - CORTECON – (90% confluency, 65% confluency) (1-2). Baseline expression levels are in TPM (transcripts per million). The raw data were reorganized and presented as heatmaps by using online BAR HeatMapper Plus software (http://bar.utoronto.ca/ntools/cgi-bin/ntools_heatmapper_plus.cgi).

**
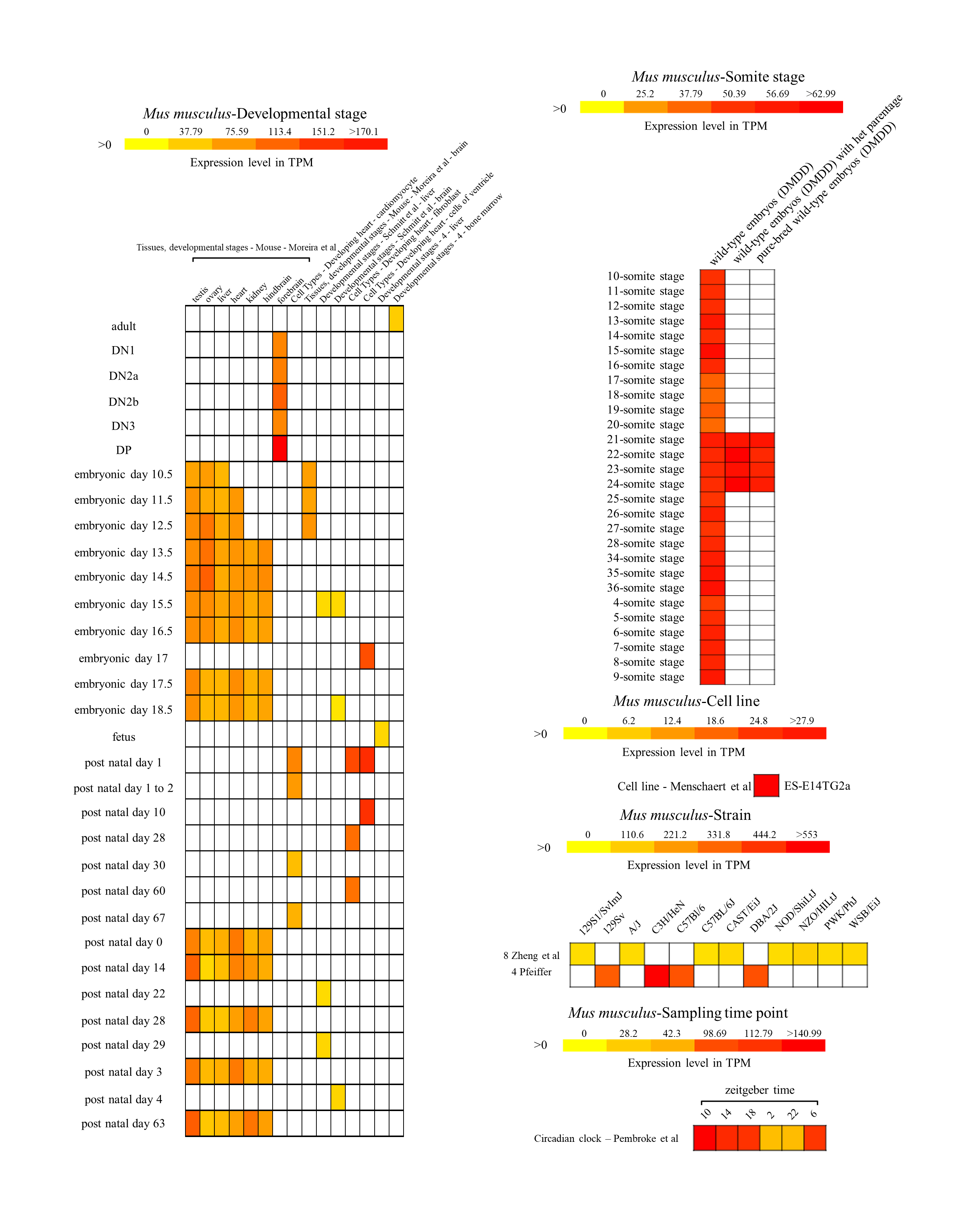
**

**Fig. S2 Expression of mouse SYF2 in various developmental stages, somite stages, cell lines, strains and sampling time points.**

Mus musculus- developmental stage No. 1-15 represent 15 data source projects, which are Tissues, developmental stages - Mouse - Moreira et al. – (testis, ovary, liver, heart, kidney, hindbrain, forebrain) (1-7), Cell Types - Developing heart – cardiomyocyte (8), Tissues, developmental stages - Mouse - Moreira et al. – brain (9), Developmental stages - Schmitt et al. – (liver, brain) (10-11), Cell Types - Developing heart – (fibroblast, cells of ventricle) (12-13), Developmental stages - 4 – (liver, bone marrow) (14-15). Mus musculus-Somite stage Nos. 1-3 represent 3 data source projects, which are wild-type embryos (DMDD) (1), wild-type embryos (DMDD) with het parentage (2), and pure-bred wild-type embryos (DMDD) (3). Mus musculus-cell line 1 represents 1 data source project, ES-E14TG2a. Mus musculus- strain Nos. 1-12 represent 12 data source project, which are 129S1/SvImJ (1), 129Sv (2), A/J (3), C3H/HeN (4), C57Bl/6 (5), C57BL/6J (6), CAST/EiJ (7), DBA/2J (8), NOD/ShiLtJ (9), NZO/HILtJ (10), PWK/PhJ (11), WSB/EiJ8 (12). Mus musculus- sampling time points 1-6 represent zeitgeber time (10, 14, 18, 2, 22, 6) (1-6). Baseline expression levels are in TPM (transcripts per million). The raw data were reorganized and presented as heatmaps by using online BAR HeatMapper Plus software (http://bar.utoronto.ca/ntools/cgi-bin/ntools_heatmapper_plus.cgi).

**
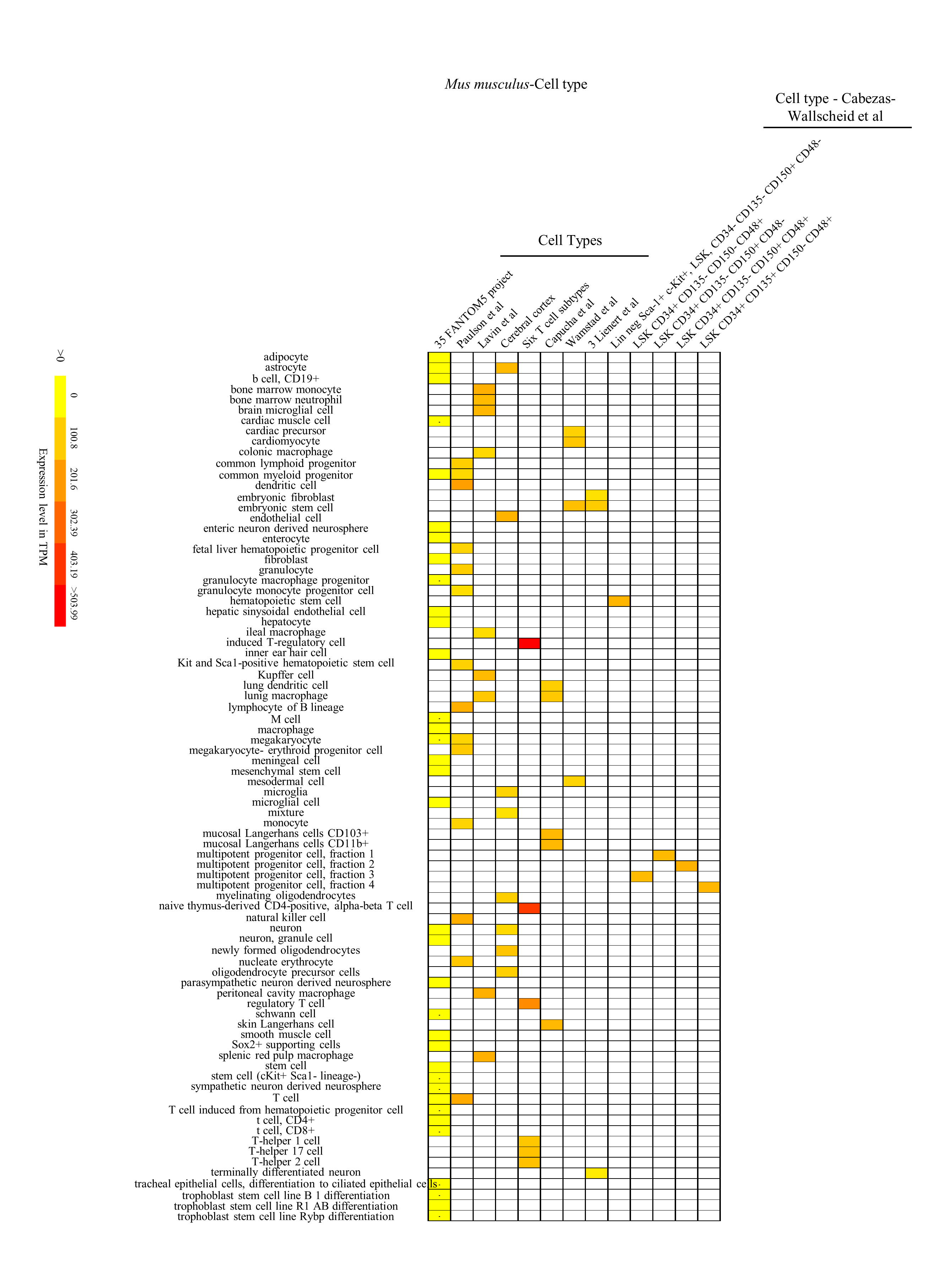
**

**Fig. S3 Expression of mouse SYF2 in various cell types.** Nos. 1-13 represent 13 data source project, which are Cell Types - (35 FANTOM5 project, Paulson et al, Lavin et al, Cerebral cortex, Six T cell subtypes, Capucha et al, Wamstad et al, 3 Lienert et al, Cabezas-Wallscheid et al. - Lin neg Sca-1+ c-Kit+, LSK, CD34- CD135- CD150+ CD48-, Cabezas-Wallscheid et al. - LSK CD34+ CD135- CD150- CD48+, Cabezas-Wallscheid et al. - LSK CD34+ CD135- CD150+ CD48-, Cabezas-Wallscheid et al. - LSK CD34+ CD135+ CD150+ CD48+, Cabezas-Wallscheid et al. - LSK CD34+ CD135+ CD150- CD48+) (1-13), respectively. Baseline expression levels are in TPM (transcripts per million). The raw data were reorganized and presented as heatmaps by using online BAR HeatMapper Plus software (http://bar.utoronto.ca/ntools/cgi-bin/ntools_heatmapper_plus.cgi).
